# Tubulin post‐translational modifications control neuronal development and functions

**DOI:** 10.1002/dneu.22774

**Published:** 2020-08-29

**Authors:** Marie‐Jo Moutin, Christophe Bosc, Leticia Peris, Annie Andrieux

**Affiliations:** ^1^ Grenoble Institut Neurosciences, University Grenoble Alpes, Inserm, U1216, CEA, CNRS Grenoble France

**Keywords:** neuro‐diseases, neuron, post‐translational modifications, tubulin, tyrosination

## Abstract

Microtubules (MTs) are an essential component of the neuronal cytoskeleton; they are involved in various aspects of neuron development, maintenance, and functions including polarization, synaptic plasticity, and transport. Neuronal MTs are highly heterogeneous due to the presence of multiple tubulin isotypes and extensive post‐translational modifications (PTMs). These PTMs—most notably detyrosination, acetylation, and polyglutamylation—have emerged as important regulators of the neuronal microtubule cytoskeleton. With this review, we summarize what is currently known about the impact of tubulin PTMs on microtubule dynamics, neuronal differentiation, plasticity, and transport as well as on brain function in normal and pathological conditions, in particular during neuro‐degeneration. The main therapeutic approaches to neuro‐diseases based on the modulation of tubulin PTMs are also summarized. Overall, the review indicates how tubulin PTMs can generate a large number of functionally specialized microtubule sub‐networks, each of which is crucial to specific neuronal features.

## 1 INTRODUCTION

Microtubules (MTs), constructed from α‐ and β‐tubulin, are one of the main components of the neuronal cytoskeleton, and constitute a critical structure for their differentiation, integrity, and function. These structures provide structural support, serve as long‐distance tracks for neuronal transport, and can participate in localized cell signaling. Tubulin in neurons is highly heterogeneous thanks to the expression of multiple tubulin isotypes, each of which is also regulated at the post‐translational level.

Tubulin can be modified by a large combination of post‐translational modifications (PTMs) such as phosphorylation, polyamination, palmitoylation, S‐nitrosylation, ubiquitylation, sumoylation, glycosylation, and methylation (Caron, Vega, Fleming, Bishop, & Solomon, 2001; Jaffrey, Erdjument‐Bromage, Ferris, Tempst, & Snyder, 2001; Ji et al., 2011; Park et al., 2016; Peters, Furlong, Asai, Harrison, & Geahlen, 1996; Rosas‐Acosta, Russell, Deyrieux, Russell, & Wilson, 2005; Srivastava & Chakrabarti, 2014; Wohlschlegel, Johnson, Reed, & Yates, 2004; Xu, Paige, & Jaffrey, 2010). These modifications have been little studied, and consequently, their functions in neurons are poorly documented. Interestingly, among the few studies available, phosphorylation of serine 172 in neuronal β tubulin was shown to be mediated by a kinase that has been linked to Down Syndrome and Autism Spectrum Disorders (Ori‐McKenney et al., 2016). In cycling cells, the same modification is reported to be performed by another kinase, cdk1 (cyclin‐dependent kinase 1) (Caudron et al., 2010; Fourest‐Lieuvin et al., 2006). This phosphorylation event regulates microtubule dynamics and neuronal function (Fourest‐Lieuvin et al., 2006; Ori‐McKenney et al., 2016), and mutation of residue 172 in humans was linked to migration defects and perturbations of axon tract formation associated with brain dysgenesis (Jaglin et al., 2009; Ori‐McKenney et al., 2016). Another modification, tubulin polyamination, which consists of the irreversible covalent binding of a polyamine to various glutamine residues on α and β‐tubulin, was shown to regulate MT stability in neurons (Song et al., 2013).

In addition to these PTMs, it has long been known that the majority of neuronal MTs are stable and functionally modified through detyrosination, acetylation, and polyglutamylation (Barra, Rodriguez, Arce, & Caputto, 1973; Hallak, Rodriguez, Barra, & Caputto, 1977; Janke et al., 2005; Paturle‐Lafanechere et al., 1991; Rogowski et al., 2009, 2010). Most of the enzymes mediating these modifications have been identified and inhibitors discovered, and these tools have been used to gain a better understanding of the roles of these PTM in neuronal functions. This chapter will thus focus on the complexity of detyrosination, acetylation, and polyglutamylation in neurons, all of which are crucial to the development and functions of these post‐mitotic cells. We start the chapter by describing the distributions of these PTM in neurons and then discuss the various elements making them crucial for neuronal function in normal and pathological conditions. The involvement of detyrosination, acetylation, and polyglutamylation in brain disorders, as well as PTM‐based therapeutic approaches, will also be considered.

## 2 TUBULIN PTMs AND MT DYNAMICS IN NEURONS

Neurons display an extremely polarized morphology, with structurally and functionally distinct compartments (the dendrites and the axon) emanating from the cell body. As a rule, dendrites are short, branched, and receive afferent information, whereas the axon is thinner and longer, and responsible for transmitting electrical signals to efferent neurons. During the early stages of development and neurite elongation, the growth cone interprets extracellular signals guiding the growth of this structure. This growth cone is an extremely labile element, mainly composed of actin and dynamic MTs. In contrast, the majority of MTs in axons and dendrites are extremely stable—with an estimated half‐life of several hours (compared to several minutes for dynamic MTs) (Baas & Black, 1990). MTs in axons and dendrites are arranged in bundles to allow their growth and maintenance. Following complete neuronal maturation, dendrites contain small actin‐rich protrusions named dendritic spines, the morphological and molecular plasticity of which play key roles in learning and memory. Dynamic MT invasions of spines appear to be associated with changes in synaptic activity, contributing significantly to dendritic spine plasticity (Dent, Merriam, & Hu, 2011; Hu, Viesselmann, Nam, Merriam, & Dent, 2008; Jaworski et al., 2009; Schatzle et al., 2018). Most neuronal MTs are non‐centrosomal, i.e., not anchored to an MT‐organizing center, and may thus have different orientations. In mature neurons, the axon contains almost only parallel plus‐end‐out MTs, whereas dendritic processes include equal numbers of plus‐ and minus‐end‐out MT orientations (Baas, Slaughter, Brown, & Black, 1991; Yau et al., 2016).

### 2.1 Detyrosination/tyrosination cycle and MT dynamics

Tyrosination was the first tubulin PTM discovered. In the 1970s, an Argentinean team observed that rat brain homogenate could incorporate tyrosine into α tubulin in a translation‐independent manner (Arce, Barra, Rodriguez, & Caputto, 1975; Arce, Rodriguez, Barra, & Caputo, 1975; Barra et al., 1973). Shortly after making this observation, the team discovered that the reaction was reversible (Hallak et al., 1977). Molecular cloning then revealed that most α‐tubulin genes encode a carboxy‐terminal tyrosine, suggesting that the cycle is initiated by a detyrosination event (Valenzuela et al., 1981). What became known as the detyrosination/tyrosination cycle was found to be controlled by the enzymatic removal of the C‐terminal tyrosine of α‐tubulin by a Tubulin Carboxypeptidase (TCP) and its re‐addition by a Tubulin Tyrosine Ligase (TTL) (Argarana, Barra, & Caputto, 1978) (although the molecular identities of these proteins remained elusive). This cycle generates two pools of α‐tubulin in cells: tyrosinated and detyrosinated pools, which have been extensively studied using specific antibodies (Figure 2a) (Aillaud et al., 2016; Kilmartin, Wright, & Milstein, 1982).

The molecular identity of TTL was solved many years after these initial discoveries through biochemical purification and cloning (Ersfeld et al., 1993; Schroder, Wehland, & Weber, 1985), and the enzyme has since been extensively studied. To date, no other physiological substrate for TTL has been identified, and the detyrosination/tyrosination cycle appears to be exclusive to α‐tubulin. TTL preferentially acts on soluble tubulin (Kreis, 1987; Webster, Gundersen, Bulinski, & Borisy, 1987), and structural studies revealed how it binds to the detyrosinated α and β subunits of the dimer (Prota et al., 2013; Szyk, Deaconescu, Piszczek, & Roll‐Mecak, 2011). In cells, TTL activity is regulated by protein kinase C phosphorylation (Idriss, 2000, 2001) and through its interaction with MAP1B (Barnat et al., 2016; Utreras et al., 2008). In vitro TTL activity was also shown to be weakened by cytoskeleton regulator stathmin (Szyk, Piszczek, & Roll‐Mecak, 2013).

Despite multiple attempts at purification over more than 40 years, the identity of TCP remained unknown until recently, when two teams simultaneously, using two distinct approaches (Aillaud et al., 2017; Nieuwenhuis et al., 2017), demonstrated that this activity is performed by vasohibins (VASH1 or VASH2) associated with a partner subunit (small vasohibin‐binding protein, SVBP). Crystal structures for both enzymatic complexes were presented in several recent studies (Adamopoulos et al., 2019; Li, Hu, Qi, Luo, & Yu, 2019; Liao et al., 2019; Liu et al., 2019; Wang et al., 2019; Zhou, Yan, Zhang, & Liu, 2019). These structures demonstrate that SVBP acts as a chaperone protein stabilizing VASH (Suzuki et al., 2010), and as a positive regulator of detyrosination activity (Wang et al., 2019). Both of the studies that identified VASH‐SVBP enzymatic complexes as TCPs (Aillaud et al., 2017; Nieuwenhuis et al., 2017) agreed that other detyrosinating enzyme(s) remain to be identified, since abundant detyrosinating activity continues to be observed after VASH knockdown in neurons, and knockout in cell lines. A recent study reported that adult mice brain missing the regulatory SVBP element still has around 60% detyrosinated tubulin (Pagnamenta et al., 2019). Notably, in neurons and the brain, tubulin isotype variability might also contribute to the pool of detyrosinated tubulin as a result of expression of the α4 tubulin isotype, which is genetically encoded without a C‐terminal tyrosine (Gu, Lewis, & Cowan, 1988; Redeker, Rossier, & Frankfurter, 1998; Strassel et al., 2019). In the adult rat brain, 15% of the α‐tubulin pool was shown to be this persistently non‐tyrosinated isotype (Redeker et al., 1998). Another α‐tubulin isotype, α8, contains C‐terminal phenylalanine instead of a tyrosine and is only expressed transiently in the brain at the onset of cortical neurogenesis. This isotype was shown very recently to interfere with tyrosination signaling (Ramos et al., 2020), but how it perturbs tyrosination and Δ2‐tubulin cleavage remains as yet unknown.

Tyrosination has been defined as a marker of dynamic MTs, as tyrosinated MTs are sensitive to the MT‐depolymerizing drug nocodazole (Baas & Black, 1990; Bre, Kreis, & Karsenti, 1987; Kreis, 1987) and are substrates for Kinesin‐13 depolymerizing motors (Peris et al., 2009). In contrast, detyrosinated MTs are resistant to disassembly by nocodazole and motor‐dependent mechanisms (Peris et al., 2009). Detyrosination has, therefore, been defined as a marker of MT stability. Unlike TTL, which acts on soluble tubulin, TCP preferentially acts on MTs (Figure 2a) (Arce, Hallak, Rodriguez, Barra, & Caputto, 1978; Bre et al., 1987; Kreis, 1987).

**FIGURE 1:**
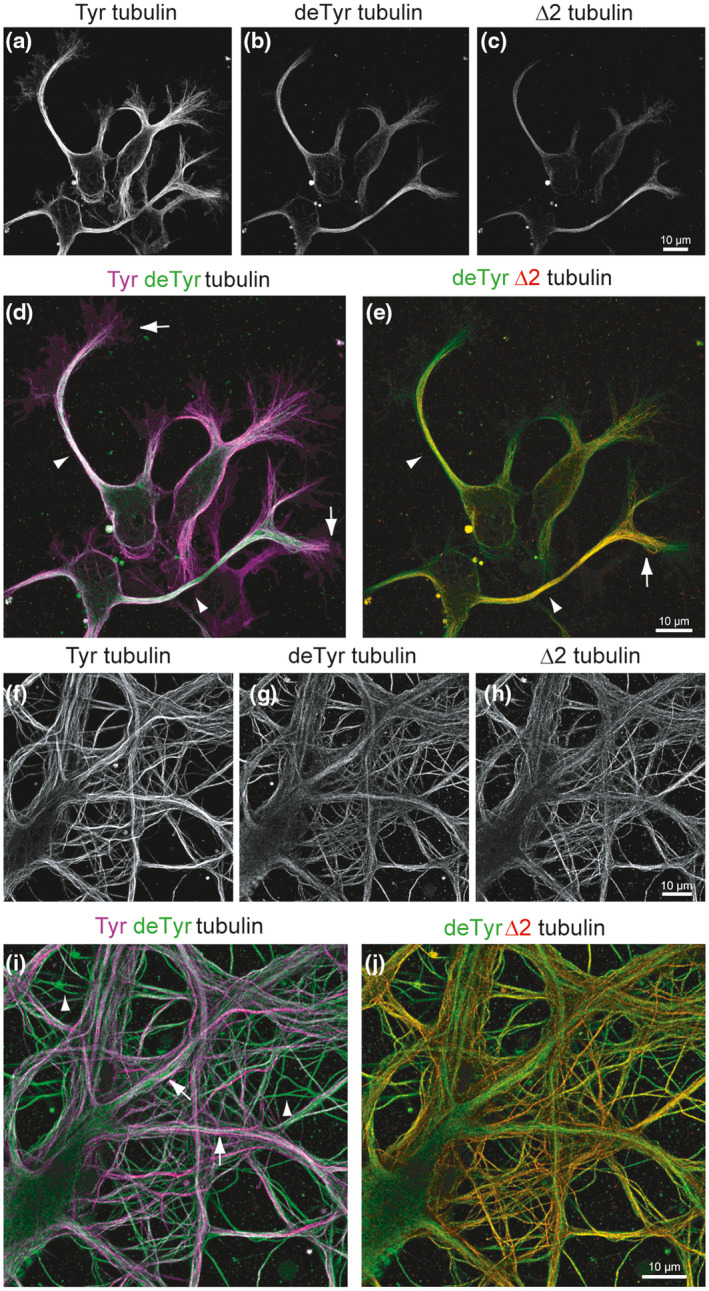
Distribution of tyrosinated (Tyr), detyrosinated (deTyr), and ∆2 tubulin in developing and mature neurons. Airyscan confocal images of hippocampal neurons after 2 (a‐e) or 18 days in culture (f‐j). Cells were labeled with anti‐Tyr, anti‐deTyr, and anti‐∆2 tubulin antibodies (Aillaud et al., 2016). Static immunostaining images of Tyr, deTyr, and ∆2 tubulin labeling were obtained with a ×63 oil‐immersion objective (1.4 NA) using an inverted confocal microscope (LSM 710, Zeiss) linked to an Airyscan detector to improve signal‐to‐noise ratio and spatial resolution. Scale bar = 10 µm. **At early developmental stages**, the majority of neuronal microtubules bear Tyr tubulin (a). DeTyr microtubules are enriched in axons (arrowheads), but are also detected in neurites (b). ∆2 tubulin is mainly enriched in axons (c). Color‐combined image of Tyr (magenta) and deTyr tubulin (green) reveals that stable deTyr microtubules are enriched in axons (arrowheads), while most dynamic Tyr microtubules are found in the growth cone (arrows) (d). The color‐combined image of deTyr (green) and ∆2 tubulin (red) reveals a different distribution of these modified tubulins inside the axon: while deTyr and ∆2 tubulin colocalize in axons (arrowheads) and at the proximal part of the axonal growth cone (arrow), deTyr tubulin extends further into neurites and growth cones (e). **In mature neurons**, dynamic Tyr microtubules are enriched in the outer part of primary large dendrites and in some thin structures that could be axons emanating from neighboring neurons (f). DeTyr microtubules are seen bundled in the inner part of large dendrites, and are also highly enriched in most thin structures (g). ∆2 tubulin is mainly present in the thin structures (h). Color‐combined image of Tyr (magenta) and deTyr tubulin (green) reveals an asymmetric pattern in large dendrites (Tyr outside and deTyr inside, arrowhead), and the enrichment of deTyr microtubules in axon‐like structures (arrow) (i). Color‐combined image of deTyr (green) and ∆2 tubulin (red) reveals a different distribution of the modified tubulins with only partial colocalization in thin axon like structures (j)

**FIGURE 2:**
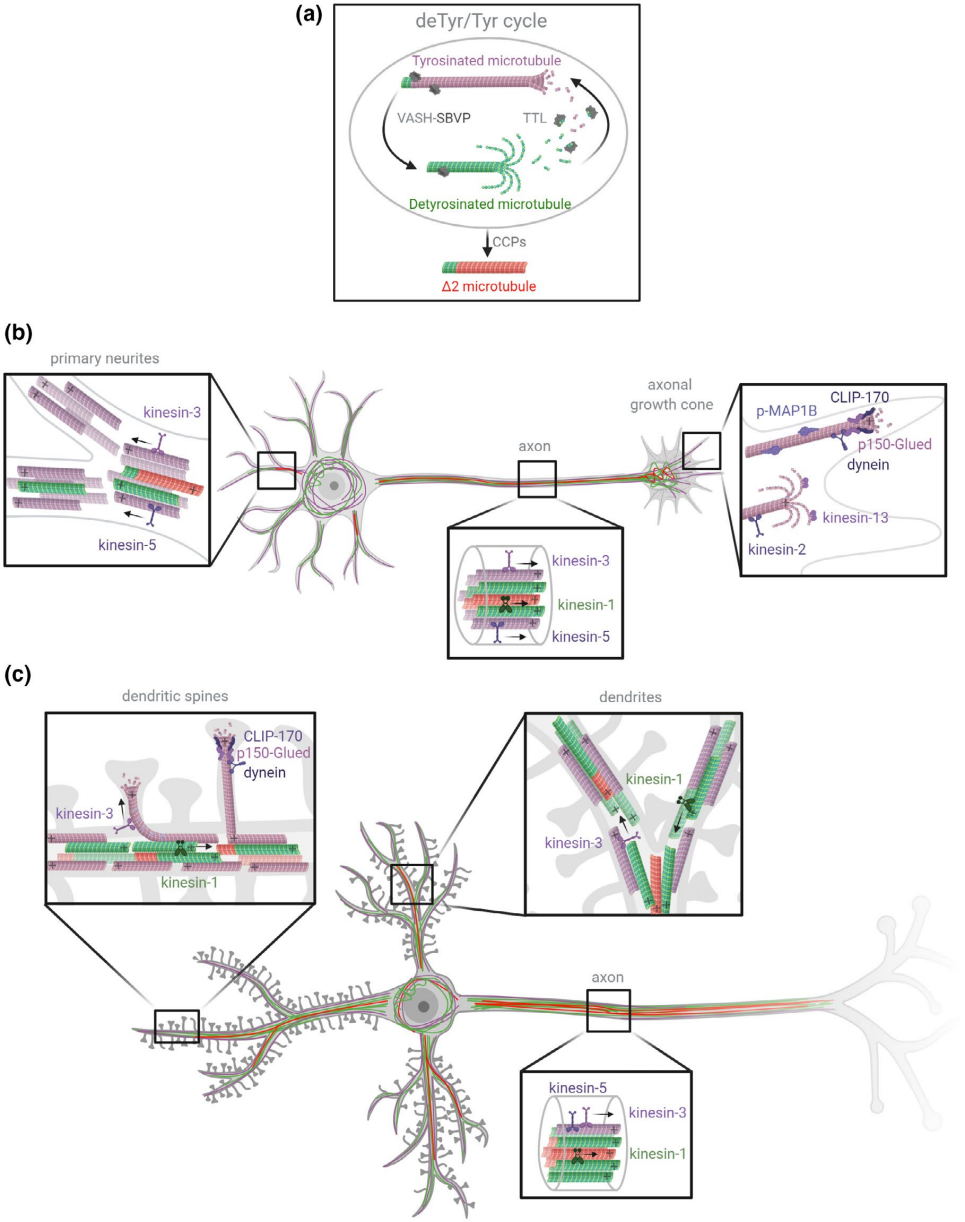
Schematic representation of the three major pools of α‐tubulin resulting from the detyrosination/tyrosination cycle in neurons, with their known effectors. (a) The tubulin cycle and Δ2 tubulin production. The VASH‐SVBP complex (VASH1 or VASH2 associated with small vasohibin‐binding protein, SVBP) removes the C‐terminal tyrosine residue of α‐tubulin incorporated in microtubules to generate detyrosinated (deTyr) microtubules (green). After detyrosinated microtubules depolymerize, tubulin tyrosin ligase (TTL) can once again add a tyrosine residue to α/β tubulin dimers. When these soluble dimers polymerize, they form new tyrosinated (Tyr) microtubules (magenta). In long‐lived detyrosinated microtubules, the penultimate glutamate residue of α‐tubulin can be removed by cytosolic carboxypeptidases (CCPs), generating microtubules composed of Δ2 tubulin (red). (b) Distribution of α‐tubulin pools (Tyr/deTyr/Δ2) in developing neurons. During the development of neurons, primary neurites contain mixed‐polarity microtubules mainly composed of Tyr tubulin (magenta). Stable deTyr microtubules (green) are found in the inner region of neurites. Some neurites also contain hyperstable ∆2 microtubules (red). The axon contains uniform polarity microtubules that are highly enriched in deTyr and ∆2 tubulin, mainly in their inner region. The proximal axonal growth cone is composed of stable deTyr and ∆2 microtubules whereas the dynamic axonal growth cone contains Tyr microtubules in its distal region. (c) Distribution of α‐tubulin pools (Tyr/deTyr/Δ2) in mature neurons. In fully developed neurons, dendrites contain mixed‐polarity microtubules composed of Tyr tubulin in their outer region, and deTyr and ∆2 tubulin in their inner region. The Tyr microtubules from dendrites can transiently enter into dendritic spines. Axonal microtubules have uniform polarity and are mainly composed of stable deTyr and ∆2 tubulin. Molecular effectors of the cycle, such as molecular motors, CAP‐Gly plus‐end proteins (CLIP‐170 and p150 Glued), and MAP1B (phosphorylated) are represented. *Figure created with* Biorender.com

Whereas axonal MTs, which are particularly stable, are highly enriched in detyrosinated tubulin, the labile MTs present in growth cones and the distal regions of neurites are predominantly tyrosinated (Figures 1d and 2b). This variation in PTM distribution reveals the functional difference between dynamic and stable MTs in neurons. Whereas neurons have stable MTs in cell extensions to support their architecture and promote efficient intracellular transport, they also require dynamic MTs within the growth cone to allow rapid reorganization of the cell cytoskeleton in response to guiding signals.

In addition to the detyrosination/tyrosination cycle, detyrosinated tubulin can be further processed by cytosolic carboxypeptidases from the deglutamylase family. These enzymes generate subtypes of α‐tubulin named ∆2 and ∆3 tubulins as they lack the final two or three C‐terminal residues (Arce et al., 1978; Paturle‐Lafanechere et al., 1991, 1994; Raybin & Flavin, 1977a, 1977b; Rogowski et al., 2010). ∆2 tubulin, which cannot be tyrosinated by TTL and is thus excluded from the tyrosination cycle, is neuron‐specific (Paturle‐Lafanechere et al., 1994; Prota et al., 2013). This form of α‐tubulin distributes in dendrites and axons, and is considered to be a marker of hyper‐stable MTs. In contrast, Δ3 tubulin, which is also neuron‐specific, appears to be a component of dynamic MTs, like tyrosinated tubulin (Aillaud et al., 2016). Specific antibodies discriminating between tyrosinated, detyrosinated and ∆2 C‐terminal α tubulin forms have been invaluable tools in the elucidation of the roles played by these modifications in cells (Kilmartin et al., 1982; Paturle, Wehland, Margolis, & Job, 1989; Paturle‐Lafanechere et al., 1991, 1994).

In mice, the loss of the tyrosination enzyme results in lethal neuronal and whole‐brain defects, whereas loss of VASH‐SVBP‐family TCPs has a less extensive impact (Erck et al., 2005; Pagnamenta et al., 2019). The phenotypes observed will be detailed in Section “Tubulin PTM roles in brain function and dysfunction”, below.

### 2.2 Tubulin acetylation and MT dynamics

Acetylated tubulin was identified and analyzed thanks to an antibody directed against the acetylated form of lysine 40 in the α‐tubulin chain. This form of tubulin was shown to be preferentially enriched in axons compared to dendrites (Falconer, Vielkind, & Brown, 1989; Piperno & Fuller, 1985). Although most known tubulin PTMs occur at the external surface of MTs, acetylation of lysine 40 mainly locates in the MT lumen. Acetylation is catalyzed by tubulin acetyltransferase αTAT1, which preferentially acts on MTs (Akella et al., 2010; Shida, Cueva, Xu, Goodman, & Nachury, 2010). The converse reaction, deacetylation, is mainly driven by histone deacetylase 6 (HDAC6) and sirtuin 2 (SIRT2), both of which are present in the cytoplasm (Hubbert et al., 2002; North, Marshall, Borra, Denu, & Verdin, 2003). Acetylated MTs have long been considered to be stable (long‐lived MTs), and indeed, recent data indicate that the relationship between acetylation and MT stabilization is causative, as acetylation alters the conformation of the flexible loop containing lysine 40, thus affecting lateral contact sites which directly contribute to MT stability (Eshun‐Wilson et al., 2019). In accordance with these observations, the disruption of the MEC‐17 (αTAT1) gene in Tetrahymena leads to more labile MTs (Akella et al., 2010). In addition to this effect, tubulin acetylation weakens lateral interactions between protofilaments, and thus enhances MT flexibility, making them more resistant to mechanical stresses (Portran, Schaedel, Xu, Thery, & Nachury, 2017). Thus, in cells, acetylation‐promoted flexibility of MTs allows them to better resist mechanical forces and lattice damage, and as a result, they are long‐lived (Xu et al., 2017).

Functional insights into the role played by tubulin acetylation in neurons have largely been obtained by the manipulation of the acetyltransferase αTAT1, or of the deacetylases SIRT2 and HDAC6.

Although none of these enzymes is vital for organism development and survival, altered tubulin acetylation has some impact on neuronal function, as detailed below.

### 2.3 Tubulin polyglutamylation and MT dynamics

Tubulin polyglutamylation consists of the progressive ATP‐dependent addition of glutamate residues on glutamates near the C‐terminus of α and β tubulins, generating amino acid side chains of various lengths. MT polymers are the preferred substrate for this complex modification catalyzed by several enzymes containing a domain related to TTL, the tubulin tyrosine ligase‐like (TTLL) enzymes (van Dijk et al., 2007; Janke et al., 2005). Among the numerous TTLL polyglutamylases (nine have been reported in humans), two are expressed at high abundance in the mammalian nervous system. One major neuronal polyglutamylase was discovered to be a multiprotein complex including the catalytic TTLL1 and the polyglutamylase subunit 1, PGs1 (Pathak, Austin, & Drummond, 2011; Regnard et al., 2003). TTLL1 is only active when included in this multiprotein complex, and shows a substrate preference toward α‐tubulin (Janke et al., 2005; van Dijk et al., 2007). The other abundant neuronal polyglutamylase is TTLL7, which is a β‐tubulin tail polyglutamylase that works as a single polypeptide (van Dijk et al., 2007). The atomic structure of TTLL7 was recently solved (Garnham et al., 2015). This enzyme is the first deglutamylase for which a structure is available. The reverse reaction, deglutamylation, was shown to be accomplished by enzymes in the cytosolic carboxypeptidase family (CCP1‐6) (Kimura et al., 2010; Natarajan, Gadadhar, Souphron, Magiera, & Janke, 2017; Rogowski et al., 2010; Tort et al., 2014), taking either polymers or soluble tubulin as substrates (Audebert et al., 1993).

In the 1990s, mass spectrometry and the specific antibody GT335 revealed tubulin polyglutamylation to be abundant within the mammalian nervous system and to represent a major event in neurons (Edde et al., 1990; Wolff, Houdayer, Chillet, de Nechaud, & Denoulet, 1994). The GT335 antibody, obtained using a synthetic peptide mimicking the structure of the polyglutamylated site of α‐tubulin, was used to extensively study the distribution of both α‐ and β‐glutamylated tubulins in cells and tissues. Sophisticated polyglutamate structures exist in brain tubulin as a result of both α‐ and γ‐linkages in glutamyl chains (Wolff et al., 1994).

At the molecular level, polyglutamylation was shown to modulate the binding of kinesins and MT‐associated proteins (MAPs) including MAP2, Tau, and MAP1B (Larcher, Boucher, Lazereg, Gros, & Denoulet, 1996). MAP binding was optimal for tubulin modified by three glutamate residues and decreased sharply when longer lateral glutamate chains were present (Bonnet et al., 2001; Regnard et al., 2003). Polyglutamylation might thus facilitate selective recruitments into distinct MT populations, hence modulating the functional properties of MTs. Polyglutamylation, specifically with long glutamate side chains, has also been linked to the increased severing of MTs by spastin‐ and katanin‐dependent mechanisms (Lacroix et al., 2010; Sharma et al., 2007; Valenstein & Roll‐Mecak, 2016; Zehr, Szyk, Szczesna, & Roll‐Mecak, 2020) which could be involved in the control of MT mass and dynamics. Glutamylated chains on tubulin were shown to be non‐linear biphasic tuners of spastin activity, becoming inhibitory beyond a certain threshold (Valenstein & Roll‐Mecak, 2016). A recent structural study revealed the essential role played by long glutamate stretches for katanin ATPase activity (Zehr et al., 2020).

A mice lacking the main neuronal polyglutamylases revealed a critical role for this modification in cilia and flagella, and displayed mild neuronal defects (Ikegami, Sato, Nakamura, Ostrowski, & Setou, 2010). Deglutamylase deficiency, in contrast, clearly associates with neurodegeneration, as detailed below.

## 3 TUBULIN PTM IMPLICATION IN NEURONAL DIFFERENTIATION

Neurons use their MT arrays as architectural elements to model their axons and dendrites. Thus, MTs are critical when seeking to establish and maintain polarization. During the early stages of neuronal development, tubulin PTMs are involved in initiating axon formation, a crucial event in breaking symmetry and establishing cell polarization.

### 3.1 Detyrosination/tyrosination cycle and neuron differentiation

The tubulin forms derived from the cycle (tyrosinated, detyrosinated, and ∆2 tubulin) change their distribution during neuronal differentiation, and these changes correlate with alterations to MT dynamics. The distribution in developing and mature hippocampal neurons is presented in Figure 1 and schematized in Figure 2.

During the early stages of neuronal development, most MTs are composed of tyrosinated tubulin (Arregui, Busciglio, Caceres, & Barra, 1991). Shortly before axonal specification, MTs from one of the immature neurites become more stable and show a small increase in tubulin detyrosination (Arregui et al., 1991; Mansfield & Gordon‐Weeks, 1991; Witte, Neukirchen, & Bradke, 2008). Axonal MTs then organize in highly stable parallel arrays with a high proportion of detyrosinated, ∆2, and acetylated subunits (Brown, Li, Slaughter, & Black, 1993; Bulinski & Gundersen, 1991; Paturle‐Lafanechere et al., 1994). The most distal region of the axon, however, is mainly composed of highly dynamic tyrosinated MTs (Ahmad, Pienkowski, & Baas, 1993; Aillaud et al., 2016) (Figures 1a‐e and 2b). A recent study indicates that phosphorylated MAP1B preferentially associates with these tyrosinated MTs of the growth cone, where it may interfere with the detyrosination process, possibly keeping MTs in a dynamic state in this region (Barnat et al., 2016).

As neurons mature, MTs continue to become more stable and to accumulate PTMs. Tyrosinated MTs remain abundant in the cell body, whereas distinct patterns of post‐translationally modified tubulin form in dendritic and axonal compartments, with dynamic tyrosinated MTs located in the outer part of the dendrite, and stable (acetylated and ∆2 tubulin bundles) in the inner part (Tas et al., 2017) (Figures 1f‐j and 2c). Dendritic spines are transiently invaded by dynamic tyrosinated MTs in an activity‐dependent manner, suggesting that MTs, in cross‐talk with the actin cytoskeleton, play an important role in synapse function and plasticity (Dent et al., 2011; Hu et al., 2008; Jaworski et al., 2009; Schatzle et al., 2018).

Alterations to the detyrosination/tyrosination cycle following the manipulation of the enzymes involved have extensive consequences on neuronal differentiation. Neurons cultured from TTL knockout mice contain no tyrosinated tubulin, and this absence of dynamic tyrosinated MTs has dramatic effects on neuronal morphogenesis. In these cells, neurite outgrowth is accelerated and erratic, and premature axonal differentiation is observed. These neurons exhibit enlarged growth cones with an abnormal invasion of detyrosinated MTs in the peripheral actin‐rich domain, and an increased proportion of neurons bearing two or more axons and supernumerary branches (Erck et al., 2005; Marcos et al., 2009). In TTL‐null cells, both axons and dendrites contain the same levels of detyrosinated MTs, suggesting that the axonal enrichment of detyrosinated MTs is a key determinant controlling neuronal polarization. At the molecular level, the phenotype of TTL‐null neurons has been linked to the inhibition of Kinesin‐13's MT‐depolymerizing activity (Homma et al., 2003; Peris et al., 2009), reduced Kinesin‐2 activity leading to MT‐destabilization (Gumy et al., 2013), decreased recruitment of Myosin IIB, and increased Rac1 activity in the growth cone (Marcos et al., 2009). In addition, binding of CAP‐Gly (Cytoskeleton‐Associated Protein‐glycine‐rich domain‐containing +TIPs, such as CLIP‐170 and p150 Glued) to the plus end of detyrosinated MTs is impaired (Nirschl, Magiera, Lazarus, Janke, & Holzbaur, 2016; Peris et al., 2006), and defective polarized trafficking of Kinesin‐1 and Kinesin‐5 motors is observed (Cai, McEwen, Martens, Meyhofer, & Verhey, 2009; Dunn et al., 2008; Kahn, Sharma, Gonzalez‐Billault, & Baas, 2015; Konishi & Setou, 2009; Liao & Gundersen, 1998).

In contrast, neurons lacking SVBP (missing both VASH1 and VASH2 detyrosinating enzymes) show high levels of tyrosinated tubulin and a partial loss of detyrosinated tubulin (Pagnamenta et al., 2019). Remaining detyrosinated pools are concentrated in axons. Young SVBP‐null neurons exhibit delayed axonal differentiation and defective axonal architecture, with an increased number of primary neurites and additional branches. After complete neuronal maturation, these neurons still have significantly more dendritic branches than wild‐type neurons. Conversely, overexpression of VASH2 in neurons causes premature axonal differentiation and, reminiscent of the phenotype observed in TTL‐null neurons, a threefold increase in the emergence of abnormal neurons bearing two axons (Wang et al., 2019).

### 3.2 Tubulin acetylation and neuron differentiation

Direct evidence of the role played by acetylated tubulin in neuronal differentiation remains sparse. Available data indicate that acetylated α‐tubulin is involved both in dendrite and axon morphogenesis. In Drosophila bearing a mutated α‐tubulin gene where lysine 40 was replaced by an arginine residue (K40R), abnormal dendrite branch refinement in sensory neurons was observed extensively branched dendrites (Jenkins, Saunders, Record, Johnson‐Schlitz, & Wildonger, 2017). Similarly, in mice, loss of α‐tubulin acetylation as a result of MEC‐17 (α‐TAT1) ablation, predisposes neurons to axon overbranching, and excessive growth (Dan et al., 2017). The contribution of acetylated tubulin to neuronal morphogenesis could be related to the modulation of αTAT1 activity by CAMSAP3 (calmodulin‐regulated spectrin‐associated protein 3), a key player in MT minus‐end dynamics. CAMSAP3 preferentially associates with nonacetylated MTs, and this interaction is important in maintaining neuronal polarity (Pongrakhananon et al., 2018). Tubulin acetylation was also recently proposed to be a downstream consequence of CYLD (Cylindromatosis tumor suppressor protein) activation, which regulates dendrite morphogenesis (Li, Sekine‐Aizawa, Ebrahimi, Tanaka, & Okabe, 2019).

### 3.3 Tubulin glutamylation and neuron differentiation

Distribution of glutamylated α and β‐tubulin varies during brain development and neuron differentiation. In young neurons, glutamylated α‐tubulin is already abundant (bearing chains of 1 to 6 Glu), whereas β‐tubulin is very poorly glutamylated. During neuronal differentiation, both α‐ and β‐glutamylated tubulins accumulate to a significant extent, and this accumulation is accompanied by a decrease in the turnover of glutamyl units on tubulin (Audebert et al., 1994; Przyborski & Cambray‐Deakin, 1997). As polyglutamylated tubulin becomes more prominent in differentiating processes in neurons, it was proposed that it is involved in neurite outgrowth through interactions with specific MAPs. In line with this hypothesis, TTLL7 polyglutamylase accumulates in MAP2‐positive dendrites and is involved in neurite outgrowth from PC12 cells (Ikegami et al., 2006).

## 4 TUBULIN PTMs CONNECTION TO NEURONAL TRANSPORT AND PLASTICITY

Neurons are highly polarized cells that depend on active intracellular transport for their morphogenesis, function, and survival. This transport, achieved by the molecular motors kinesin and dynein is controlled by both the orientation of MTs and tubulin PTMs. In axons, where MTs are oriented plus‐end‐out, transport has been intensively studied. Thanks to these investigations, we know that anterograde axonal transport is mediated by kinesins, whereas retrograde axonal transport is mediated by dyneins. These two transport modes supply the distal synapses with newly synthesized proteins and allow clearance of damaged proteins. Several studies reported that the molecular motors were modulated by tubulin PTMs, and this could be used by the cells as a means to specify the localization of motors in different areas of the neuron, and thus to direct trafficking. Recent mathematical modeling experiments indicated that even a slight modulation of motor kinetics could be sufficient to target the motors to specific locations along the MT array (Iniguez & Allard, 2017).

### 4.1 Detyrosination/tyrosination cycle and neuronal transport

Kinesin‐1 was found to be predominantly present on stable detyrosinated MTs in axons, and tyrosinated tubulin was thus proposed to be a repulsive signal for this motor, restricting its somatodendritic localization (Dunn et al., 2008; Hammond et al., 2010; Konishi & Setou, 2009). Indeed, loss of tyrosination in neurons leads to the redistribution of axonal Kinesin‐1 to dendrites (Cai et al., 2009; Konishi & Setou, 2009) and to the formation of multiple axons (Erck et al., 2005). No preference of Kinesin‐1 for recombinant detyrosinated MTs was observed in vitro (Sirajuddin, Rice, & Vale, 2014). The preferential location of Kinesin‐1 on stable axonal MTs might be controlled by a combination of tubulin modifications including acetylation (Hammond et al., 2010), as recently demonstrated by tracking of motors on MTs using super‐resolution techniques (Tas et al., 2017). In this study, Tas and colleagues showed that dynamic tyrosinated MTs and stable acetylated/Δ2 MTs form non‐overlapping bundles of opposite orientation in dendrites. This organization enhances the specific recruitment of Kinesin‐1 motors to stable MT bundles, and thus biases its transport directionality, from dendrites toward the axon.

In contrast, Kinesin‐3 and Kinesin‐5 motors prefer tyrosinated neuronal MTs, and they can thus enter both axons and dendrites (Guardia, Farias, Jia, Pu, & Bonifacino, 2016; Kahn et al., 2015; Tas et al., 2017). The initiation of retrograde transport by dynein‐dynactin complexes in the distal region of the axon was recently shown to be partly controlled by α‐tubulin tyrosination (Nirschl et al., 2016). Tyrosinated tubulin allows the spatial regulation of the transport initiation event along with CLIP‐170 phosphorylation, which modulates spatiotemporal patterns. However, the C‐terminal tyrosine does not affect the motility of the molecular motor complex (McKenney, Huynh, Vale, & Sirajuddin, 2016; Nirschl et al., 2016). Notably, in degenerative neurons containing mutated spastin, focal impairment of retrograde transport was observed in the distal axonal region characterized by a transition between detyrosinated and tyrosinated α‐tubulin (Tarrade et al., 2006).

In addition, an intriguing link between detyrosination and transport was recently revealed with the finding that Kinesin‐1 affects MT detyrosination by targeting TCP enzymes onto specific MTs (Yasuda, Clatterbuck‐Soper, Jackrel, Shorter, & Mili, 2017).

Finally, abnormal ratios of tyrosinated/detyrosinated tubulin in neurons most probably induce synaptic plasticity defects and abnormal dendritic spine homeostasis, as proposed for non‐functional VASH‐SVBP complexes (Iqbal et al., 2019; Pagnamenta et al., 2019). However, additional in vitro and in vivo analyses of neuronal morphology and spine integrity will be needed to obtain a full picture of how synaptic functioning depends on the detyrosination/tyrosination cycle.

### 4.2 Tubulin acetylation and neuronal transport

Impaired α‐tubulin acetylation results in reduced binding of molecular motors to MTs, thereby leading to reduced neuronal transport of vesicles (Fukushima, Furuta, Hidaka, Moriyama, & Tsujiuchi, 2009; Reed et al., 2006). It appears that tubulin acetylation does not directly affect Kinesin‐1 motility but rather that, as acetylated MTs are bundled, it enhances kinesin run lengths (Balabanian, Berger, & Hendricks, 2017; Kaul, Soppina, & Verhey, 2014; Walter, Beranek, Fischermeier, & Diez, 2012). In Drosophila, modulation of α‐TAT1 activity by p27 (Kip1) was shown to be critical for axonal transport of vesicles and organelles (Morelli et al., 2018). Inhibition of SIRT2 and the resulting increase in acetylated tubulin cause increased autophagic vesicular traffic and cargo clearance (Esteves et al., 2019). In a Drosophila model, mutant forms of Leucine‐rich repeat kinase 2 (LRRK2) cause the accumulation of deacetylated MTs associated with axonal transport deficits in primary neurons. In addition, restoring normal levels of acetylated tubulin, following knockdown of the deacetylases HDAC6 and Sirt2, rescues axonal transport (Godena et al., 2014).

### 4.3 Tubulin glutamylation and neuronal transport

Polyglutamylated tubulin has been suggested to serve as a molecular traffic signal for synaptic transmission‐related axonal transport and to be modulated by synaptic transmission. Mice lacking functional PGs1 (ROSA22 mutants), a protein associated with the main neuronal polyglutamylase TTLL1, exhibit a loss of polyglutamylated α‐tubulin within neurons, associated with decreased MT binding affinity of KIF1A—a molecular motor of the Kinesin‐3 family crucial for axonal transport (Ikegami et al., 2007). This alteration to transport affects the proportion of synaptic vesicles in the CA1 region of the hippocampus in these mice and alters their synaptic transmission. In addition, increased neuronal activity (through GlyR activity blockade) facilitates tubulin polyglutamylation, which then reduces Kinesin‐1 motor protein mobility and cargo delivery to neurites (Maas et al., 2009). These effects suggest that synaptic transmission could regulate MT‐dependent cargo delivery by modulating polyglutamylation. At the molecular level, a single‐molecule imaging study recently suggested a mechanism for the regulation of Kinesin‐3 motility on MT involving pauses mediated by interactions between a specific region on the motor (K‐loop) and the polyglutamylated C‐terminal tails of MT (Lessard et al., 2019). Neuronal, and specifically axonal, transport can also be sensitive to polyglutamylation as a result of spastin‐mediated severing: a change in the polyglutamylated surface of MTs could modulate the rate of severing (Lacroix et al., 2010; Valenstein & Roll‐Mecak, 2016), leading to alterations to the MT tracks.

## 5 TUBULIN PTM ROLES IN BRAIN FUNCTION AND DYSFUNCTION

The significance of tubulin PTMs for neurons and brain tissue first emerged from mice models in which the various enzymes responsible for tubulin modification were knocked out. Current molecular understanding suggests that imbalanced levels of tubulin PTMs occur in a range of brain disorders/pathologies, including neurodegenerative conditions.

### 5.1 Detyrosination/tyrosination cycle in brain function/dysfunction

The physiological significance of the tubulin detyrosination cycle has been clearly demonstrated in mice models. In knockout mice, perturbed levels of α‐tubulin C‐terminal tyrosination mainly lead to altered brain morphology. TTL knockout mice die just after birth as a result of the disorganization of their neuronal networks (Erck et al., 2005). They display a blurred organization of the brain neocortex layers and a disrupted cortico‐thalamic loop. No obvious malformation of other organs has been detected in these newborn mice. In a recent study, brain abnormalities were also observed in SVBP knockout mice (Pagnamenta et al., 2019), corresponding to the double knockout of VASH1 and VASH2 detyrosinating enzymes (Aillaud et al., 2017; Nieuwenhuis et al., 2017). Morphological analysis by anatomical MRI revealed microcephaly and a considerable reduction in several white matter tracts in SVBP‐null brains, which display a dramatic accumulation of tyrosinated tubulin and a reduction of detyrosinated tubulin. The incomplete loss of detyrosinated tubulin in the absence of SVBP is most probably the result of the expression of the detyrosinated α4‐tubulin isotype together with the presence of other detyrosinating enzymes in brain (Aillaud et al., 2017; Nieuwenhuis et al., 2017). Very interestingly, human consanguineous families with several individuals bearing biallelic inactivation of SVBP as a result of truncating variants have been reported (Iqbal et al., 2019; Pagnamenta et al., 2019). Individuals carrying the double mutation show brain abnormalities with microcephaly, intellectual disability, and delayed gross motor and speech development. Interestingly, Ramos and colleagues very recently demonstrated that the specific α8 tubulin isotype (bearing phenylalanine instead of a tyrosine at the C‐terminus) tunes the detyrosination cycle and regulates the differentiation of radial glial progenitors in the developing mice cortex (Ramos et al., 2020). Thus, perturbation of the detyrosination cycle may lead to neurodevelopmental disease.

Alteration of the detyrosination cycle has also been proposed to be involved in other brain dysfunctions. In hypothyroidism in rats, brain detyrosinating enzymes were found to be impaired in the developing thyroid‐deficient cerebellum (Poddar & Sarkar, 1993). In phenylketonuria, a Phe residue was shown to be incorporated in place of terminal tyrosine on neuronal tubulin, altering its MT dynamics and architecture (Ditamo, Dentesano, Purro, Arce, & Bisig, 2016). In neurodegenerative processes in general, levels of detyrosinated/tyrosinated tubulin in neurons and brain have been shown to be modified and could be early markers of disease development (Gonatas, Stieber, & Gonatas, 2006; Jackson, Gruner, Qin, & Tourtellotte, 2014; Pianu, Lefort, Thuiliere, Tabourier, & Bartolini, 2014; Zhang et al., 2015).

Overall, data regarding detyrosination/tyrosination indicated a role for this PTM in brain development and connectivity, as well as in neuronal plasticity.

### 5.2 Tubulin acetylation in brain function/dysfunction

In preclinical models, abnormal levels of acetylated tubulin resulted in brain dysfunctions. In Drosophila as well as in Caenorhabditis elegans, mutation of α‐TAT1 orthologs has no effect on neuronal morphogenesis, but results in impaired mechanical sensitivity in sensory neurons and abnormal behavioral responses to touch and vibration stimuli (Davenport et al., 2014; Yan et al., 2018). Similarly, mice lacking α‐TAT1 in sensory neurons display a profoundly reduced ability to detect mechanical stimuli as they are insensitive to mechanical touch and pain. This loss of mechanosensitivity correlates with a decrease in cellular elasticity (increased cellular stiffness) (Morley et al., 2016). In addition, the organization of the dentate gyrus, septum, and striatum in mice lacking α‐TAT1 were reported to be altered and associated with enlarged lateral ventricles in the forebrain (Kim, Li, Ghorbani, You, & Yang, 2013; Li, Jayabal, et al., 2019).

Overall, data on tubulin acetylation indicate a major role for this PTM in mechanosensitivity.

### 5.3 Tubulin glutamylation in brain function/dysfunction

Perturbed levels of polyglutamylated tubulin lead to neurodegeneration. The well‐known Purkinje‐cell degeneration (pcd) mice carries a mutation in CCP1 deglutamylase and accumulates abnormally glutamylated tubulin in the brain regions where degeneration is observed. This discovery highlights the crucial role played by polyglutamylation in neuronal survival (Munoz‐Castaneda et al., 2018; Rogowski et al., 2010). Interestingly, degeneration of CCP1‐deficient neurons can be rescued by knockout of the major brain polyglutamylase TTLL1 (Magiera et al., 2018). Controlling the length of the polyglutamate side chains on tubulin is critical for central nervous system neurons, but also for peripheral nerve and spinal motor neurons (Rogowski et al., 2010; Shashi et al., 2018; Zhou et al., 2018). At the molecular level, the excessive polyglutamylation generated by the CCP1 mutation was shown to decrease neuronal transport by an unknown mechanism that does not involve spastin severing (Magiera et al., 2018). Unlike CCP1, CCP5 is not essential for neuronal survival in mice (Giordano et al., 2019; Wu, Wei, & Morgan, 2017).

Dysregulated tubulin polyglutamylation was also recently directly associated with human brain diseases. Three independent studies revealed biallelic inactivation of CCP1 to be causative for infantile‐onset neurodegeneration (Karakaya et al., 2019; Shashi et al., 2018; Sheffer et al., 2019). The disorder mainly affects the cerebellum, spinal motor neurons, and peripheral nerves. Glutamylation is the main regulator of the MT severing enzyme spastin (Lacroix et al., 2010; Valenstein & Roll‐Mecak, 2016), which is mutated in the most frequent form of hereditary spastic paraplegia, a human disease characterized by lower limb weakness (Hazan et al., 1999). Alteration of polyglutamylation patterns might affect severing by spastin, and therefore MT mass and dynamics, in affected neurons. By studying primary neurons exposed to toxic Aβ, Zempel, and colleagues (Zempel et al., 2013) also proposed a role for Tau mis‐sorting. Their hypothesis is that TTLL6 polyglutamylase mis‐localization and increased spastin severing lead to perturbed MT‐based traffic of mitochondria in Alzheimer's disease and other tauopathies.

Overall, data relating to polyglutamylation suggest a role for this PTM in neuron survival.

## 6 TUBULIN PTMs IN NEURO‐REGENERATION

Axonal regeneration is an essential part of rebuilding functional connections between injured neurons and their targets. Neurons in the peripheral nervous system have an intrinsic regeneration capacity. Upon injury, they activate a pro‐regenerative program that allows axon regeneration and functional recovery. One of the key mechanisms of growth cone formation and axonal regeneration is the regulation of MT assemblies and their dynamic properties (Erturk, Hellal, Enes, & Bradke, 2007; Hur & Saijilafu, 2012).

### 6.1 Detyrosination/tyrosination cycle and neuro‐regeneration

The tyrosination state of α‐tubulin was proposed to be involved in the axonal regeneration process many years ago (Mullins, Hargreaves, Li, Dahlstrom, & McLean, 1994). More recently, Song and colleagues (Song, Cho, Watt, & Cavalli, 2015) showed that TTL is required to increase the levels of tyrosinated α‐tubulin at the site of axon injury, and thus that it plays a significant role in recovery. Indeed, down‐regulation of TTL impaired retrograde organelle transport and delayed the activation of the pro‐regenerative program. In another study, decreasing detyrosination using parthenolide was shown to boost the nerve regeneration process; this positive effect of parthenolide could be counteracted by taxol (Gobrecht et al., 2016). Consequently, modulation of the detyrosination/tyrosination cycle in favor of tyrosination appears to stimulate neuronal regeneration and was proposed as a strategy to treat nerve damage. At the molecular level, this effect could be explained by robust recruitment of CLIP‐170 to the tyrosinated MTs, promoting dynein‐driven retrograde transport in axons (Nirschl et al., 2016), which could stimulate the transport of injury signals from the site of a lesion back to the cell soma. The efficiency of retrograde transport is crucial for the physiology of neurons and is specifically involved in their survival and regeneration (Rishal & Fainzilber, 2014). Kinesin‐13 depolymerizing motors, which are crucial to the dynamic characteristics of MTs, could also be involved at injury sites. In addition, KIF3C Kinesin‐2 could be implicated, as it is exclusively expressed in the nervous system during differentiation and after injury and as it exhibits a greater affinity for tyrosine MTs in vitro. This motor was proposed to function as an MT‐destabilizing factor to regulate the dynamic state of MTs (Gumy et al., 2013). The Kinesin‐2 family member KIF3C thus regulates MT dynamics and is required for axon growth and regeneration.

### 6.2 Acetylation and neuro‐regeneration

A gradient of tubulin deacetylation follows axon injury in the peripheral nervous system. This gradient is at least partly driven by HDAC5 which was identified as a novel injury‐regulated tubulin deacetylase, playing an essential role in the growth cone dynamics of regenerating axons (Cho & Cavalli, 2012). At the time of the study, it was unclear whether tubulin acetylation was required for the regeneration program, but recent work indicates that part of the inhibitory blockades preventing axon regrowth was due to a lack of α‐TAT1 activity (Wong et al., 2018). Accordingly, restoration of a normal level of acetylated tubulin, either pharmacologically or following lentiviral‐based transfection of the α‐TAT1 gene, re‐establish neurite growth (Wong et al., 2018). Thus, it appears that a precise balance between tubulin deacetylation and acetylation is required for optimal axon regeneration, with deacetylated and acetylated tubulin possibly having a beneficial effect in early and late phases, respectively.

## 7 TUBULIN PTMs AS TARGETS FOR THERAPEUTIC APPROACHES

In several neurodegenerative conditions, including both preclinical models and human samples, variations of modified tubulin or PTM enzyme levels have been reported (Cartelli et al., 2013; Dompierre et al., 2007; Maxwell et al., 2011; Vu, Akatsu, Hashizume, Setou, & Ikegami, 2017; Zempel et al., 2013; Zhang et al., 2014, 2015). These deficits in post‐translationally modified tubulin have stimulated the search for new therapeutic strategies mainly pointing to acetylated tubulin. Indeed, most pharmacological approaches aim to inhibit the major tubulin deacetylase HDAC6, or occasionally other deacetylases (sirtuins). These approaches have now been developed to treat a myriad of neuro‐diseases. Potent HDAC inhibitors are available, such as Tricostatin A, Tubastatin A, and ACY‐1215. However, it is important to note that the modulation of HDAC might not be restricted to tubulin (Pandey et al., 2007) as HDAC has a large number of other substrates. In vitro studies provided evidence that stimulating tubulin acetyltransferase activity might be another possible therapeutic avenue, but we currently lack active molecules.

The effects of tubulin deacetylase modulation in a panel of preclinical models mimicking pathological conditions are summarized below.

**Memory and synaptic plasticity** were shown to be enhanced using HDAC inhibitors through the expression of specific genes crucial for memory consolidation (Vecsey et al., 2007). A subset of patients suffering from **human immunodeficiency virus‐1** (HIV) displays neurocognitive disorders associated with synaptodendritic simplifications. In cellular models of the disease, HDAC6 inhibitors alleviated neurite shortening and axonal transport defects, and prevented cell death (Wenzel et al., 2019). **Rett syndrome** (RTT) is a neurodevelopmental disorder caused by loss‐of‐function mutations in the transcriptional modulator methyl‐CpG‐binding protein 2 (Mecp2). In a mice model of the disease, the HDAC6 inhibitor was found to increase the speed of BDNF vesicles and to restore activity‐dependent BDNF release (Xu, Kozikowski, & Pozzo‐Miller, 2014). In addition, more recently, using induced Pluripotent Stem Cells (iPSCs) derived from patients with various Mecp2 mutations, a significantly decreased level of acetylated α‐tubulin was observed, it could be reverted by treatment with HDAC6 inhibitors (Landucci et al., 2018). **Amyotrophic lateral sclerosis (ALS)** is a rapid progressive fatal neurodegenerative disorder affecting peripheral nerves with a selective loss of motor neurons; it is at least partly caused by severe axonal transport deficits. Mutations in more than 50 genes are thought to cause ALS. This genetic heterogeneity has prevented the development of specific therapies. The possibility that HDAC6 could serve as a therapeutic target has, however, been assessed in several preclinical models. At first, using the superoxide dismutase 1 (SOD1) mice model, genetic inhibition of HDAC6 was found to prevent motor axon deficits (Taes et al., 2013). More recently, using the glycyl‐tRNA synthetase (GlyRS) model, HDAC6 inhibition was shown to restore axonal transport deficits, especially for mitochondria, to rescue nerve conduction, and to improve motor function (Benoy et al., 2018; Mo et al., 2018). Similarly, axonal transport deficits observed in motor neurons derived from iPSC from patients with ALS and FUS (fused in sarcoma) protein mutations were alleviated by HDAC6 inhibition (Guo et al., 2017). **In Parkinson's disease**, the efficacy of HDAC inhibition was shown in the Leucine‐rich repeat kinase 2 (LRRK2)‐deficient fly (Godena et al., 2014) and in the 6‐OHDA induced dopaminergic injury model (Jian et al., 2017). Similarly, sirtuin inhibitors were shown to protect against dopaminergic cell death in a Drosophila model of Parkinson's disease, although the direct implication of tubulin was not proven (Outeiro et al., 2007). **In Huntington's disease,** defects in MT‐based transport and in autophagic flux were reverted using HDAC inhibitors (Dompierre et al., 2007; Guedes‐Dias et al., 2015). **In Alzheimer's disease**, using a Drosophila model and a genetic screen, an HDAC6 mutation was found to repair tau‐induced neuronal defects (Xiong et al., 2013). In addition, HDAC6 inhibitors rescued cognitive deficits in mice by reducing the load of amyloid‐beta and hyperphosphorylated tau, suggesting increased autophagic clearance (Zhang et al., 2014). Following **ischemic stroke**, inhibition of HDAC6 protects hippocampal cells against mitochondria‐mediated apoptosis (Chang et al., 2019) and promotes the maturation of the dendritic arborization of new‐born neurons (Sheu et al., 2019). However, as it has multiple substrates, the effects of HDAC6 inhibitors might not be restricted to the modification of tubulin acetylation.

**A significant step toward the development of new treatments** for neurodegenerative and cognitive disorders related to tubulin PTMs will be the investigation of drugs modulating tubulin tyrosination and polyglutamylation levels. Interestingly, the detyrosination/tyrosination cycle might be a good target since it exclusively affects tubulin. Indirect modulation of tyrosinated tubulin levels has been associated with beneficial effects in several neuro‐pathological conditions and with various kinds of pharmacological compounds, including fluoxetine, ADNP‐derived peptide, IgM directed against gangliosides, and Icariin, an ingredient in the medicinal herb Epimedium brevicornum Maxim (Bianchi et al., 2009; Li, Ho, Chen, & Hsiao, 2016; Oz, Ivashko‐Pachima, & Gozes, 2012; Xu et al., 2015). The relatively recent discovery of the identity of the enzymes responsible for detyrosination and glutamylation, i.e., TCPs (VASH‐SVBP complexes), TTLLs, and CCPs, means that it will now be possible to search for direct inhibitors or activators. For the modulation of tyrosinated/detyrosinated tubulin levels in diseases, searches for specific inhibitors/activators might be inspired by data obtained from cellular models. TCP can be inhibited by parthenolide (Fonrose et al., 2007), leading to a beneficial effect on nerve regeneration (Gobrecht et al., 2016). This compound exhibits a low specific profile, as it targets a large number of other substrates with possible side effects for brain functions (Herrera et al., 2005; Materazzi et al., 2013; Schwarz, Bloom, Castro, Pagan, & Jimenez‐Rivera, 2011). Other compounds such as TPCK and epoY, have also been shown to modulate tubulin tyrosination in neurons (Aillaud et al., 2017; Wang et al., 2019).

The discovery of neuro specific activators or inhibitors of PTM enzymes will be of definite interest for the future treatment of neurodegenerative diseases, cognitive defects, and to improve neuronal regeneration following injuries or stroke.

## 8 CONCLUSIONS, PERSPECTIVES

Almost all enzymes altering detyrosination, acetylation, and polyglutamylation levels have now been identified, and animal models are available. However, an important question in this area remains: what is/are the last enzymatic actor(s) in the detyrosination process?

Although tubulin PTM occurs throughout the body (and in essentially all cells), many phenotypes associated with impaired or lost function are observed in the brain and neurons.

A future major challenge will be to understand the localized role(s) of each modification, or group of modifications, in the various regions of the neuronal space. Any attempt to elucidate these roles will require visualization at the microtubule level to acquire images of the complex MT organization in neurons, both during development and after final maturation. For this purpose, advanced approaches based on expansion microscopy combined with super‐resolution techniques should be applied to neuronal cells where MTs are generally stacked together. The ability to visualize individual MT should answer the unsolved questions with regard to co‐existence or segregation of the modifications along single MT and will allow potential crosstalk between PTMs to be assessed. To fulfill specific cellular functions, MT can be connected to other cytoskeletal structures, such as actin and neurofilaments. These connections, and how tubulin PTMs might modulate them, directly or through protein partners, must also be examined.

Tubulin PTMs are most probably under tight control. It will be a further challenge to understand how the diverse neuronal tubulin‐modifying enzymes are regulated in neurons at the subcellular level. Biochemical studies of the enzymes, and identification of potential protein partners and/or regulators will be of major importance. In vitro reconstitution experiments with purified tubulin, individual recombinant tubulin isotypes, and mixtures of isotypes will help researchers to figure out how each PTM is regulated as well as providing details on the crosstalk between PTMs. These experiments will help to answer the following questions: does a modifying enzyme recognize specific tubulin isotypes (or associated partners)? Does one modification affect the occurrence of another? A panel of in vitro methods including single‐molecule experiments and high‐resolution techniques will help us to understand how tubulin modifications are regulated in the more complex cellular situation.

All this evidence will undoubtedly provide us with increasing knowledge of the signaling cascades regulating the various modifications, and of the molecular mechanisms controlled by tubulin PTMs in neurons.

Finally, as abnormal tubulin PTM patterns lead to neuronal defects, the identification of small molecules modulating the activities of tubulin‐modifying enzymes could lead to the development of innovative drugs for the treatment of neuro‐diseases including neurodegenerative disorders and psychiatric diseases, as well as potentially promoting neuro‐regeneration.

## CONFLICT OF INTEREST

The authors declare no conflict of interest.
